# Herpes Simplex Virus Type 1 (HSV-1) Meningoencephalitis in an Immunocompetent Young Adolescent Female

**DOI:** 10.7759/cureus.26703

**Published:** 2022-07-09

**Authors:** Monica Garcia, Florentina Litra

**Affiliations:** 1 Pediatrics, University of Florida, Pensacola, USA; 2 Pediatrics, Ascension Sacred Heart, Pensacola, USA

**Keywords:** pediatric seizure, acute encephalitis, viral meningitis, hsv meningitis, hsv-1, viral meningoencephalitis, herpes simplex virus type 1

## Abstract

The case that we report occurred in a previously healthy, fully immunized 13-year-old female presenting with severe unilateral headaches and generalized tonic-clonic seizures and subsequently diagnosed with herpes simplex virus type 1 (HSV-1) meningoencephalitis. The patient was successfully treated with acyclovir and seizures were controlled with valproic acid. The HSV meningoencephalitis has high morbidity and mortality. Our case highlights one of the severe presentations of HSV meningoencephalitis in young adolescents that can manifest with headaches, fever, seizures, focal neurologic signs, and altered mental status. We also highlight the need for a thorough workup among pediatric providers in the emergency and inpatient departments to avoid delays in diagnosis that can lead to poor outcomes.

## Introduction

Meningoencephalitis caused by herpes simplex virus (HSV) is part of the central nervous system (CNS) disorders that can arise from a primary or recurrent HSV infection and are associated with high morbidity and mortality. Clinically, they manifest with headaches, fever, seizures, focal neurologic signs, and altered mental status. Herpes simplex encephalitis (HSE) is one of the most common causes of sporadic fatal encephalitis worldwide, about one-fifth of cases occurring in the pediatric population.

## Case presentation

A previously healthy 13-year-old, fully immunized female was admitted to the inpatient pediatric medical unit after a witnessed seizure before arrival that was described as “whole body shaking with tongue biting”. Her father reported hearing a strange noise in the patient’s room and witnessed generalized body shaking associated with diaphoresis and perioral cyanosis. When emergency medical services (EMS) arrived on the scene, the patient was in a postictal confused state and en route to the children's hospital had an episode of bowel incontinence. 

Before seizure activity, the patient had been complaining of unilateral temporal headache for five days with associated nausea and vomiting. During initial history taking on admission, it was revealed that the patient had multiple emergency room visits within the last five days for temporal headaches for which she received a cocktail of non-steroidal anti-inflammatory drugs, antiemetic and antihistaminic medications, with some relief of her symptoms. At home, they tried multiple over-the-counter headache relief medications, with no relief of symptoms. The patient had described her headaches as an “aching pain” and on initial admission rated it an 8/10 on the pain scale after she recovered from the postictal state. 

On initial examination, her exam was significant for a tongue laceration, photophobia, pupils were equal, round, and reactive to light, extraocular muscles intact, the fundoscopic exam was normal, neurologically the patient appeared at baseline, with no focal deficits, alert and oriented to person, place and time, cranial nerves II-XII grossly intact, sensation and strength intact throughout. 

Initial blood work (Table 1) was significant for WBC 12.2 K/uL (range 4.0-11.0 K/uL), 78.7% neutrophil predominance and absolute neutrophils of 9.6 K/uL (range 1.6-8.5 K/uL), complete metabolic panel was within normal limits.  

**Table 1:** Abnormal diagnostic lab results HSV: Herpes simplex virus, PCR: Polymerase chain reaction, NAAT: Nucleic acid amplification test

Diagnostic Lab	Patients Value	Range
White Blood Cells	12.2 K/uL	4.0-11.0 K/uL
Hemoglobin	12.5 g/dL	12-15.4 g/dL
Hematocrit	35.6%	35-45%
Platelets	243 K/uL	150-400 K/uL
Neutrophils	78.7%	40-70%
Lymphocytes	14.1%	30-60%
Absolute Neutrophils	9.6 K/uL	1.6-8.5 K/uL
Absolute Lymphocytes	1.70 K/uL	0.5-4.80 K/uL
Coagulation Studies		
PT (prothrombin time)	14.3 seconds	11.6-14 seconds
INR (international normalized ratio)	1.1	>4.0 critical high
PTT (partial thromboplastin time)	31.9 seconds	23-40 seconds
Complete Metabolic Panel		
Sodium	133 mmol/L	136-145 mmol/L
Potassium	3.9 mmol/L	3.4-4.7 mmol/L
Chloride	101 mmol/L	98-107 mmol/L
CO2	21 mmol/L	20-30 mmol/L
BUN	7 mg/dL	7-19 mg/dL
Creatinine	0.66 mg/dL	0.57-0.8 mg/dL
Glucose	100 mg/dL	70-99 mg/dL
Calcium	9.3 mg/dL	8.2-10.7 mg/dL
Albumin	3.6 gm/dL	3.8-5.4 gm/dL
ALT	9 Intl Units/L	11-22 Intl Units/L
AST	11 Intl Units/L	13-26 Intl Units/L
Alk Phos	187 Intl Units/L	62-280 Intl Units/L
Magnesium	2 mg/dL	1.6-2.6 mg/dL
Phosphate	3 mg/dL	2.3-4.7 mg/dL
CRP	0.07 mg/dL	<0.09 mg/dL
Cerebrospinal Fluid Studies		
White Blood Cells	480/cumm	0-6 /cumm
Red Blood cells	10/cumm	0 /cumm
Lymphocytes	93%	0%
Monocytes	4%	0%
Protein	82.7 mg/dL	15-40 mg/dL
Glucose	60 mg/dL	40-70 mg/dL
Opening Pressure	23 cm H2O	9-21 cm H2O
HSV-1 NAAT	Positive	Positive, Negative
HSV-2 NAAT	Negative	Positive, Negative
Enterovirus NAAT	Negative	Positive, Negative
Repeat Cerebrospinal Fluid Studies		
WBC	64/cumm	0-6 /cumm
Red Blood cells	72/cumm	0 /cumm
Lymphocytes	96%	0%
Monocytes	3%	0%
Opening Pressure	23 cm H2O	9-21 cm H2O
HSV-1 NAAT	Negative	Positive, Negative
HSV-2 NAAT	Negative	Positive, Negative

Differential diagnosis 

Our patient’s initial presentation of seizures, mental status changes, diaphoretic, peri-oral cyanosis, and bowel incontinence following a week of temporal headaches was suggestive of new-onset focal seizures. However, the reasoning for new-onset focal seizures ranged from space-occupying brain lesions to normal pressure hydrocephalus, migraine with aura, tension headache, subarachnoid hemorrhage, and bacterial vs viral meningoencephalitis. 

Diagnosis 

**Figure 1:**
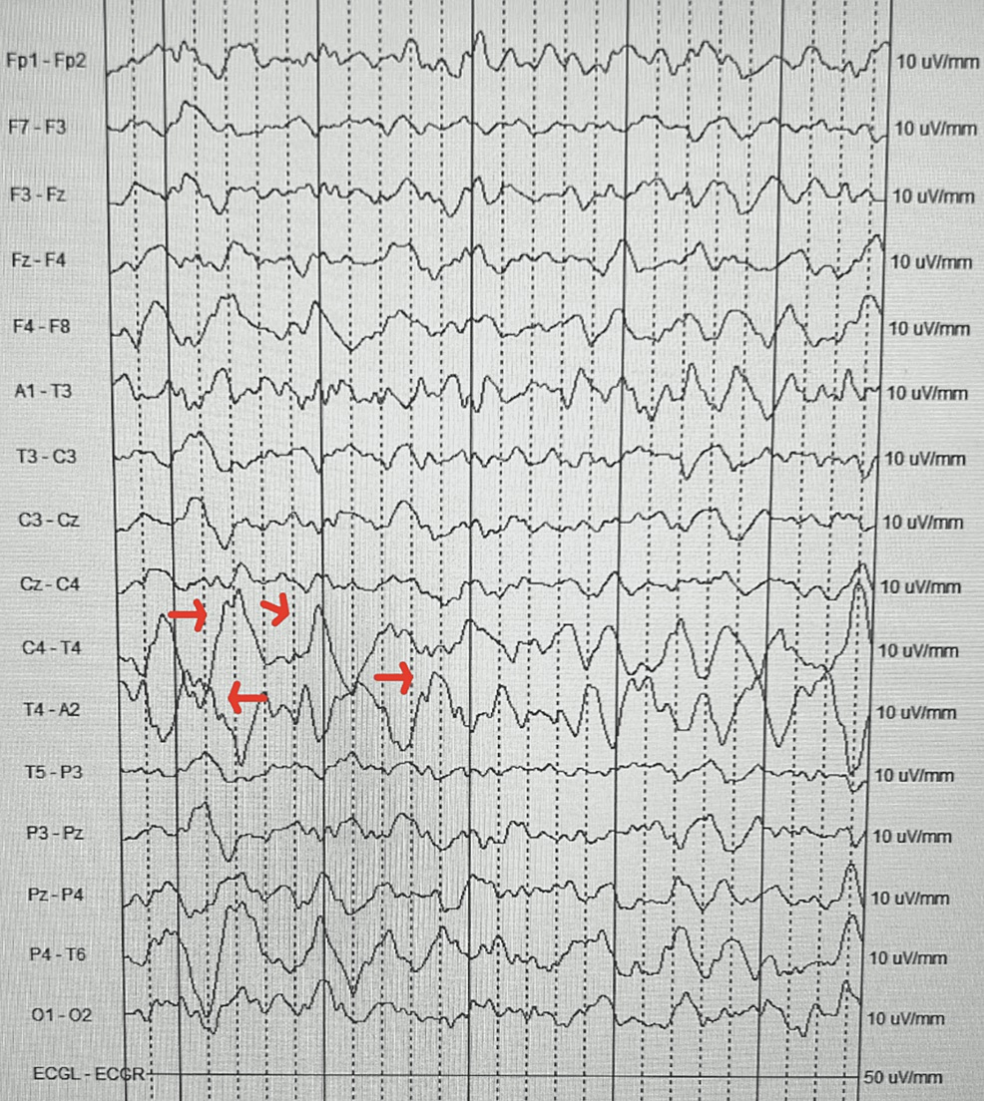
EEG at presentation Electroencephalogram (EEG) at patient presentation showing focal right-sided temporal spikes in C4-T4 and T4-A2 (red arrows).

**Figure 2:**
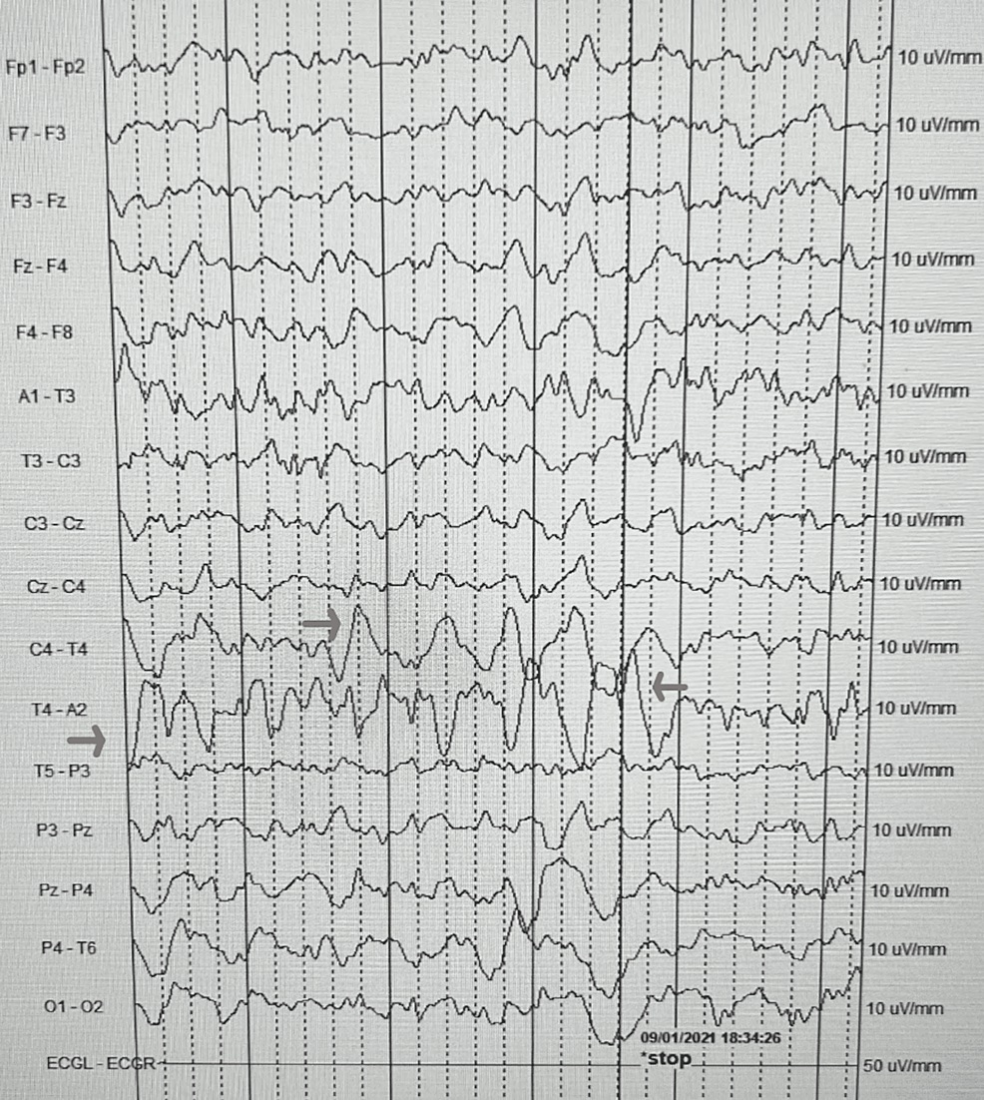
EEG at presentation with the end of the seizure Electroencephalogram (EEG) at patient presentation showing the end of the seizure in the right of the temporal lobe in C4-T4 and T4-A2 (red arrows).

**Figure 3:**
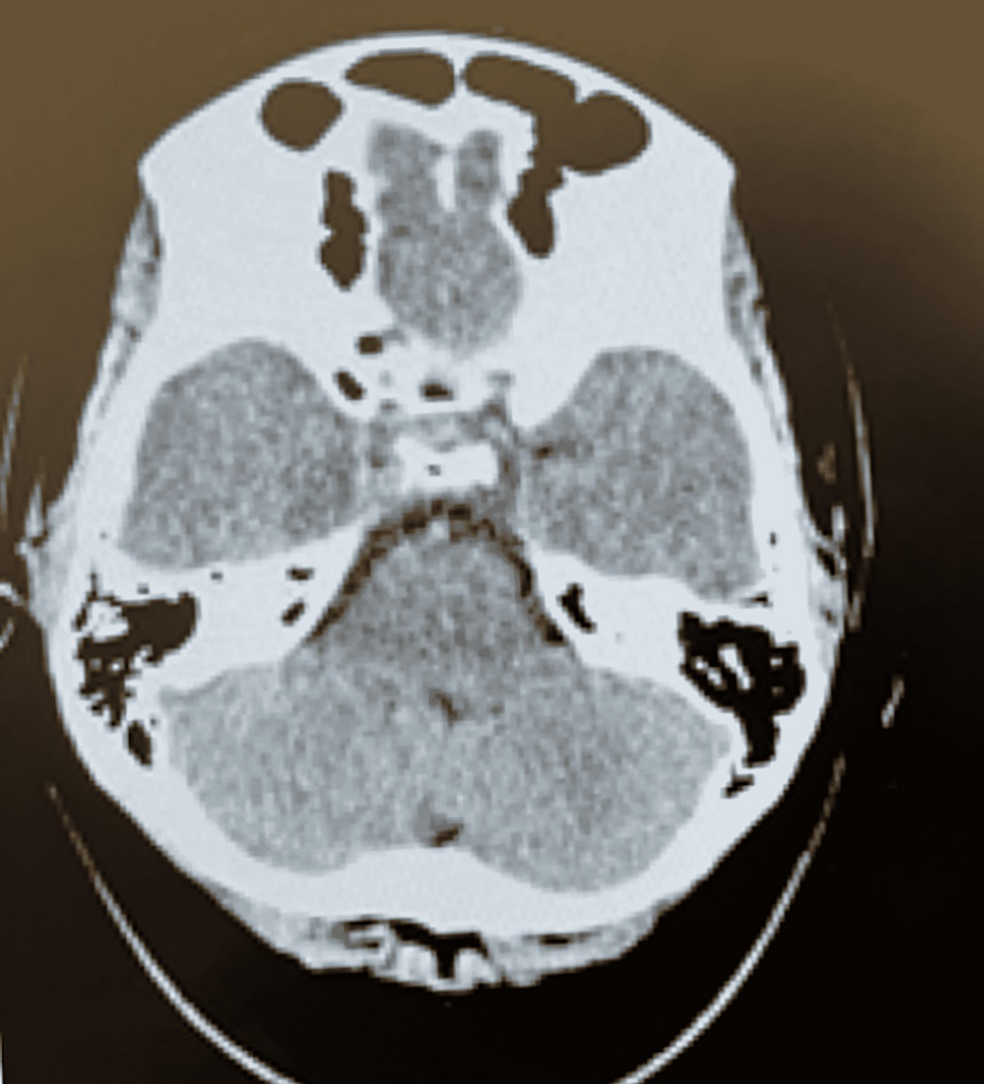
CT of the brain without contrast The axial view shows normal gray and white matter in the brain parenchyma with no evidence of cerebral injury, infarct, or intracranial hemorrhage.

On admission to the pediatric medical unit, the patient was given a loading dose of IV levetiracetam and continued twice a day maintenance dose. She was then placed on prolonged video encephalogram monitoring until she received imaging studies that night. Within one hour of being placed on an electroencephalogram (EEG), the pediatric neurologist noted focal right-sided temporal lobe abnormalities (Figure 1 and Figure 2). Overnight, the inpatient team was called to the bedside after the patient became febrile (Tmax 40.2 C), and had tachycardia and hypertension. Upon evaluation of the patient, it was noted she was only oriented to self and hard to arouse unless she was shaken awake. A STAT CT of the brain without contrast was obtained that was unremarkable (Figure 3). It was unclear if the altered mental status was from a viral/bacterial encephalitis or from a postictal seizure state or an effect of antiepileptic drugs. Due to the EEG showing spikes over the right temporal lobe, HSV encephalitis was highly suspected, and IV acyclovir was started empirically. A lumbar puncture was performed by interventional radiology under sedation the next day, due to large body habitus. Cerebrospinal fluid (CSF) studies seen above in Table 1 were significant for nucleic acid amplification test (NAAT) that was positive for herpes simplex virus type 1 (HSV-1), WBC 480/cumm (range 0-6/cumm), with 93% lymphocytes and protein of 82.7 mg/dL (range 15-40 mg/dL), and normal glucose level. The opening pressure was mildly elevated at 23 cm H2O (9 to 21 cm H2O). The CSF cultures did not show any bacterial aerobic/anaerobic growth after 48 hours. 

Patient course 

**Figure 4:**
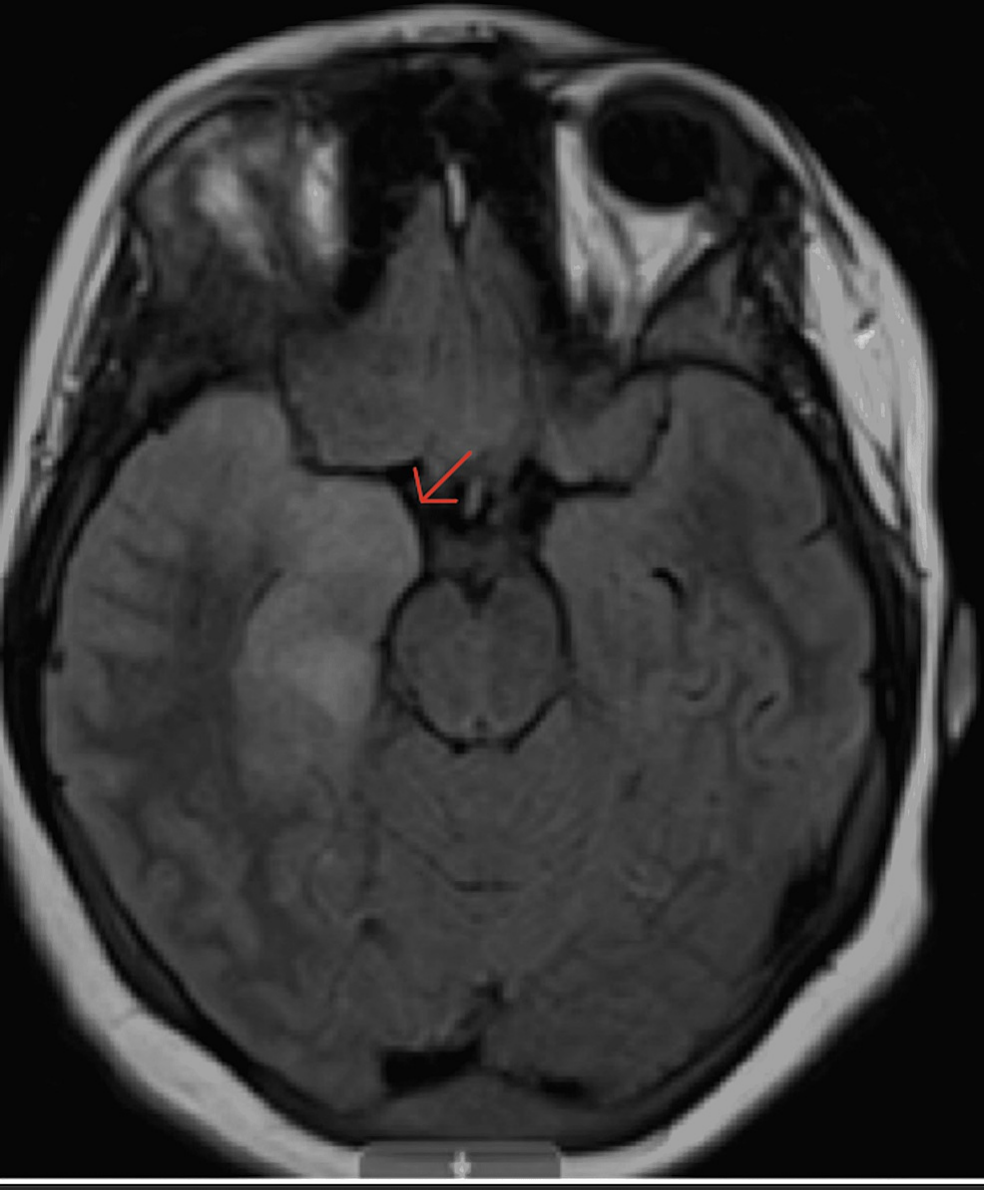
MRI of the brain without contrast in a T1-weighted axial view The axial view shows an enlarged right mesial and lateral temporal lobes with abnormal signal intensity (red arrow).

**Figure 5:**
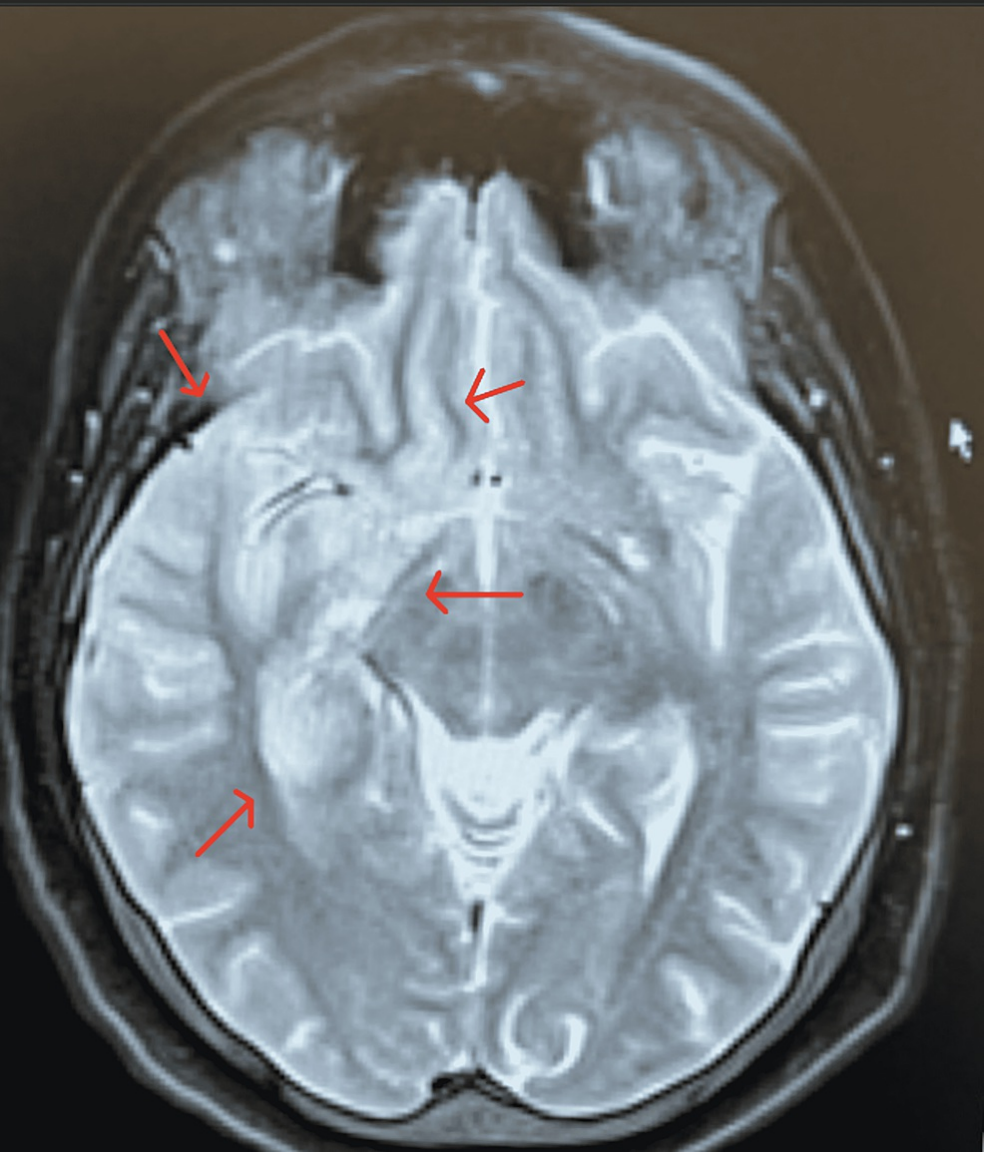
MRI of the brain with contrast in a T2-weighted axial view The axial view shows increased signal intensity and diffusion restriction is seen in the anteromedial aspect of the right temporal lobe, insular cortex, and right hippocampus (red arrows). There is a loss of gray-white differentiation in the medial aspect of the right temporal lobe.

The patient in this case completed a 21-day course of IV acyclovir while inpatient. Throughout her hospitalization, the patient complained of severe headaches, which were difficult to control with ketorolac (stopped due to acute kidney injury), hydrocodone-acetaminophen, and amitriptyline. On day three of hospitalization patient developed right eye visual changes described as "seeing double, needing to keep the eye closed to focus on things". This diplopia raised concerns prompting a repeat MRI of the brain with and without contrast (Figure 4 and Figure 5) which showed an enlarged right mesial and right lateral temporal lobe with an abnormal signal intensity that was consistent with herpes simplex encephalitis (HSE). Weekly ophthalmology exams were advised due to the persistence of these symptoms with the initial examination showing optic disc edema and esotropia.  

**Figure 6:**
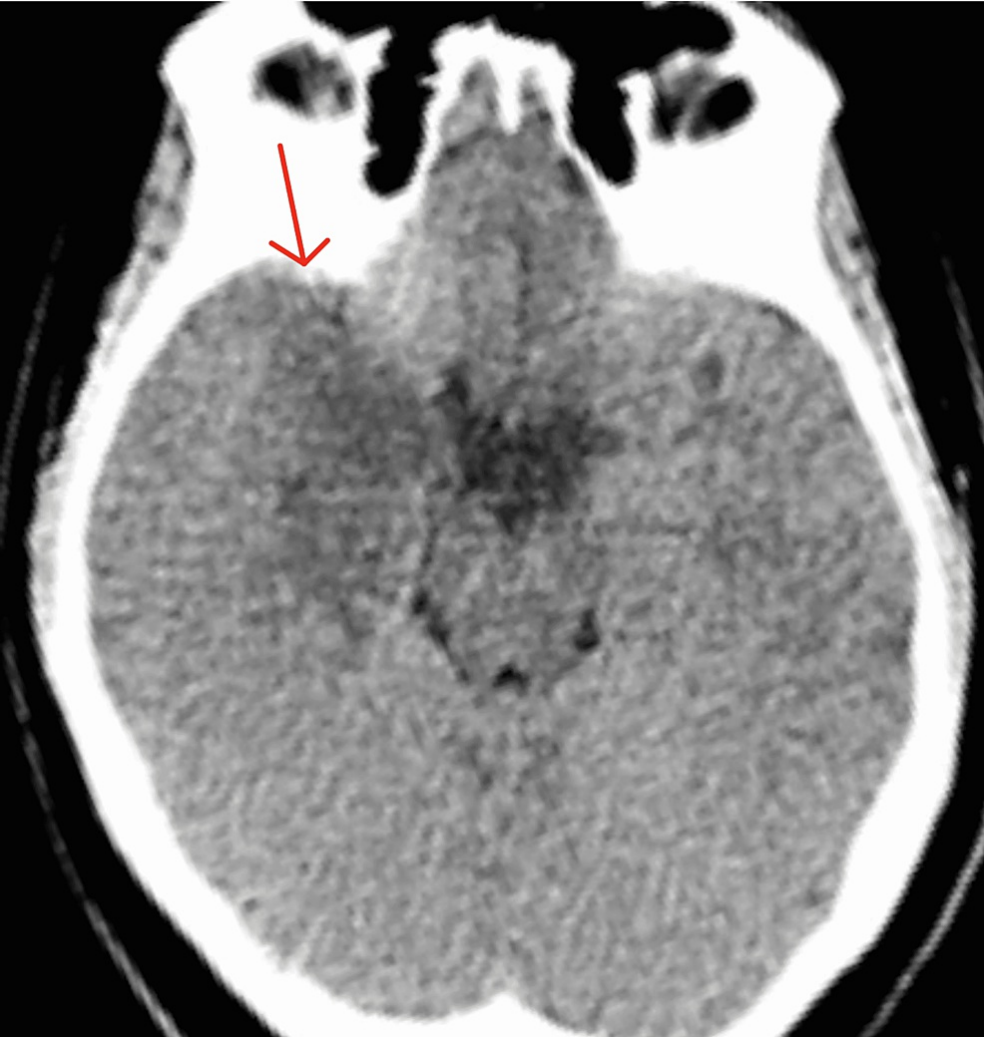
Repeat CT brain without contrast CT brain without contrast shows a new ill-defined hypoattenuation intermixed with bandlike curvilinear hyper attenuation in the right medial temporal lobe (red arrows).

The following day the patient developed multiple episodes of emesis, bradycardia, and headache concerning elevated intracranial pressures due to worsening cerebral edema. The patient had a repeat CT brain that showed mild right cortical sulcal effacement and decreased fluid distention of the right lateral ventricle compared to the previous CT brain, which was consistent with mild mass effect from the right-sided inflammation (Figure 6). Due to these findings, the patient was given 48 hours of IV dexamethasone to help decrease cerebral edema and alleviate headaches. Repeat EEG showed resolution of right temporal lobe abnormalities. 

**Figure 7:**
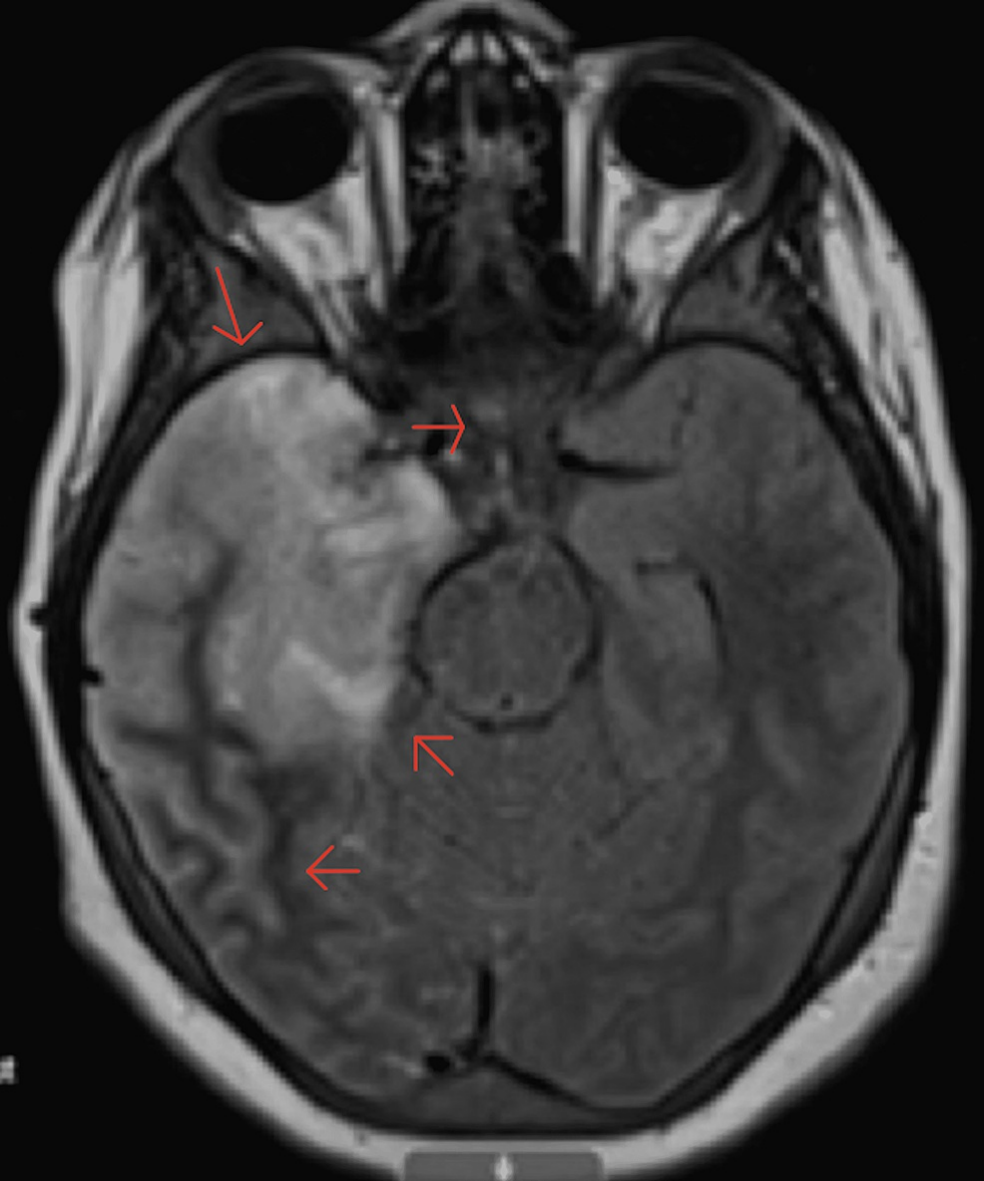
MRI brain without contrast in a T1-weighted axial view MRI brain without contrast in a T1-weighted axial view shows hemorrhagic transformation (red arrows) in the right mesial temporal lobe.

**Figure 8:**
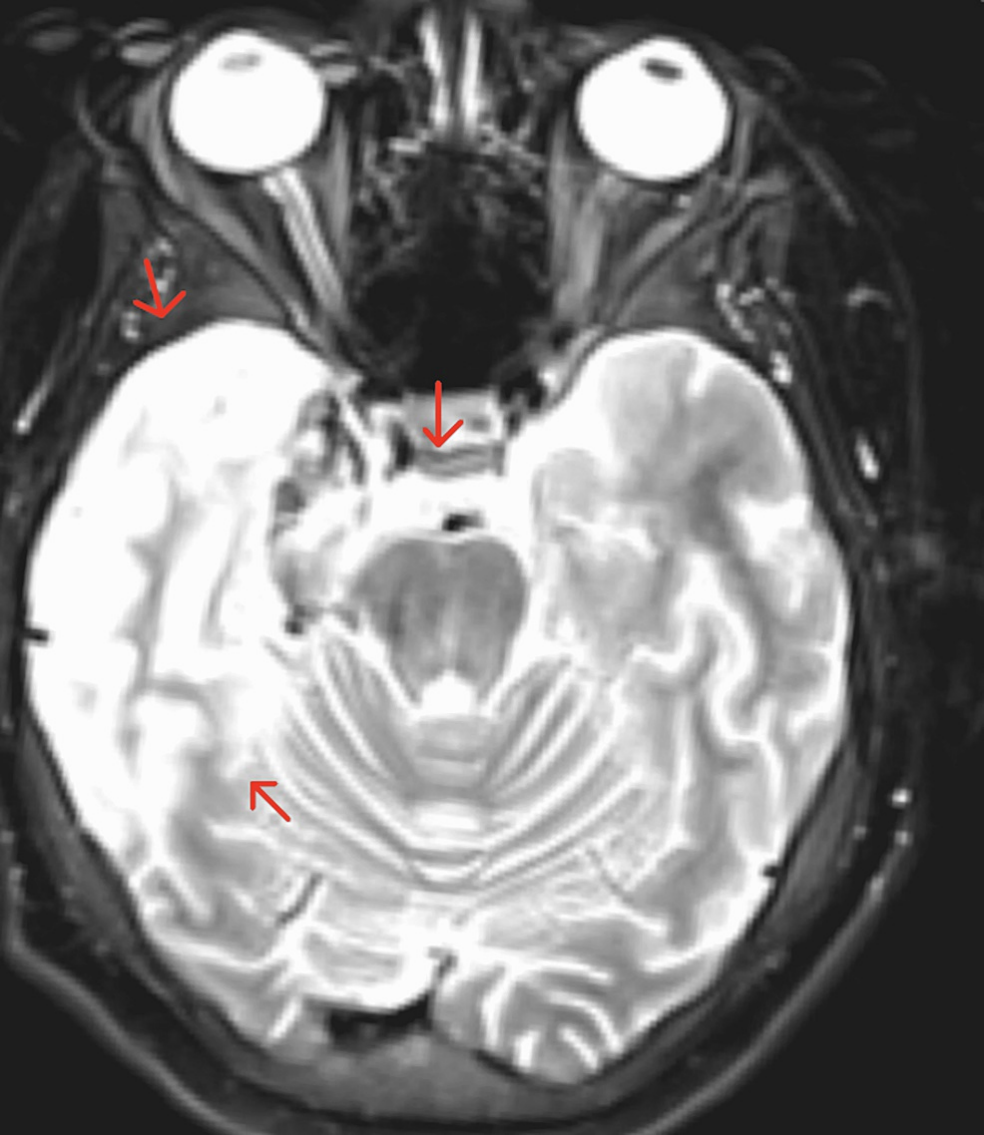
MRI brain with contrast in a T2-weighted axial view The axial view shows contrast hemorrhagic transformation with a signal alteration involving the right limbic system, right fornix, and right optic radiation. Mild effacement of the right lateral ventricle and minimal leftward midline shift.

By day seven the patient had continued headaches, intermittent confusion, and depressed mental status, and the patient had a follow-up MRI of the brain that showed worsening cerebral edema on the right anterior and mesial temporal lobe and now hemorrhagic transformation in the right mesial temporal lobe (Figure 7 and Figure 8). Coagulation studies seen above in Table 1 were performed showing stable prothrombin time (PT) of 14.3 seconds (range 11.6-14 seconds), international normalized ratio (INR) of 1.1 (range >4.0 critical high), and partial thromboplastin time (PTT) of 31.9 seconds (range 23-40 seconds) with a complete blood count (CBC) showing platelets of 257K/uL (range 150-400 K/uL). Due to vital sign changes showing concerns for increased intracranial pressure pediatric neurosurgery team was consulted and recommended that the patient’s systolic blood pressures remain between 70 mmHg and 140 mmHg to decrease the risk of further elevation of cranial pressure and possible herniation. Infectious disease also recommended a loading dose of 16 mg of dexamethasone and then 4 mg every six hours for four days. 

**Figure 9:**
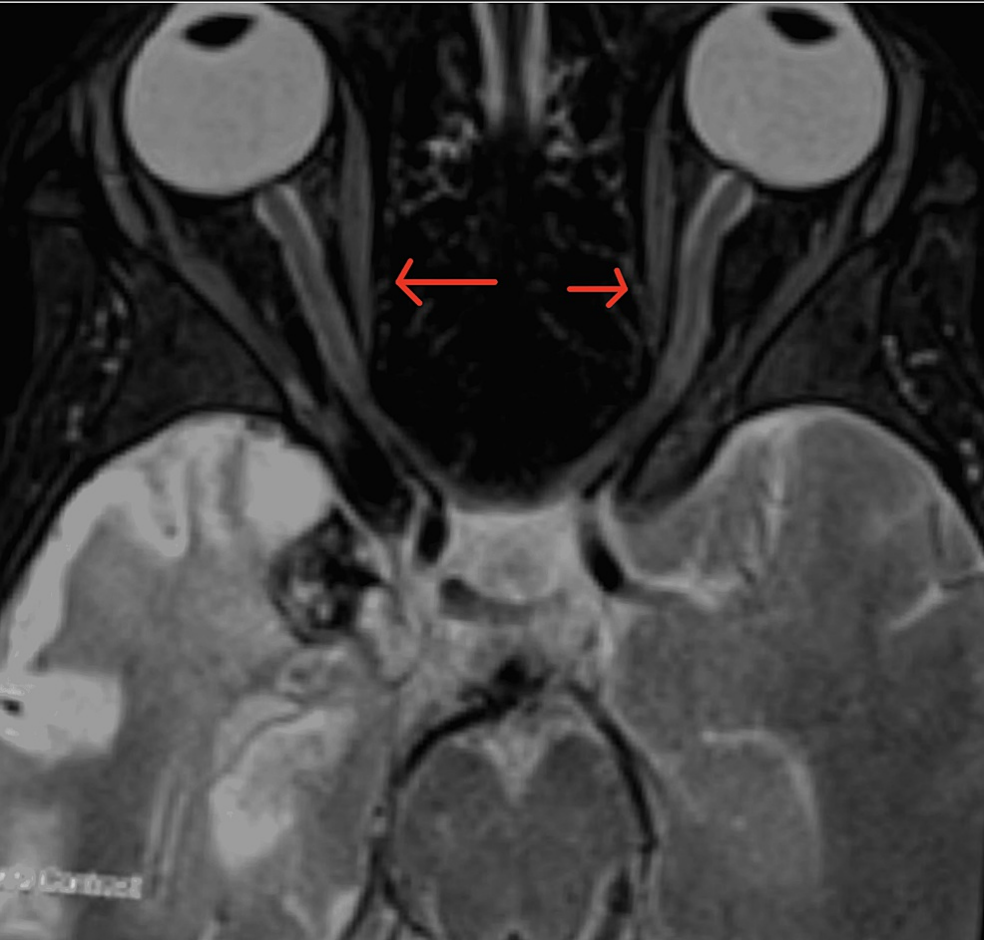
MRI brain without contrast focused on orbits, T1-weighted, axial view The axial view shows bilateral ocular globes and extra-ocular muscles that are normal.

**Figure 10:**
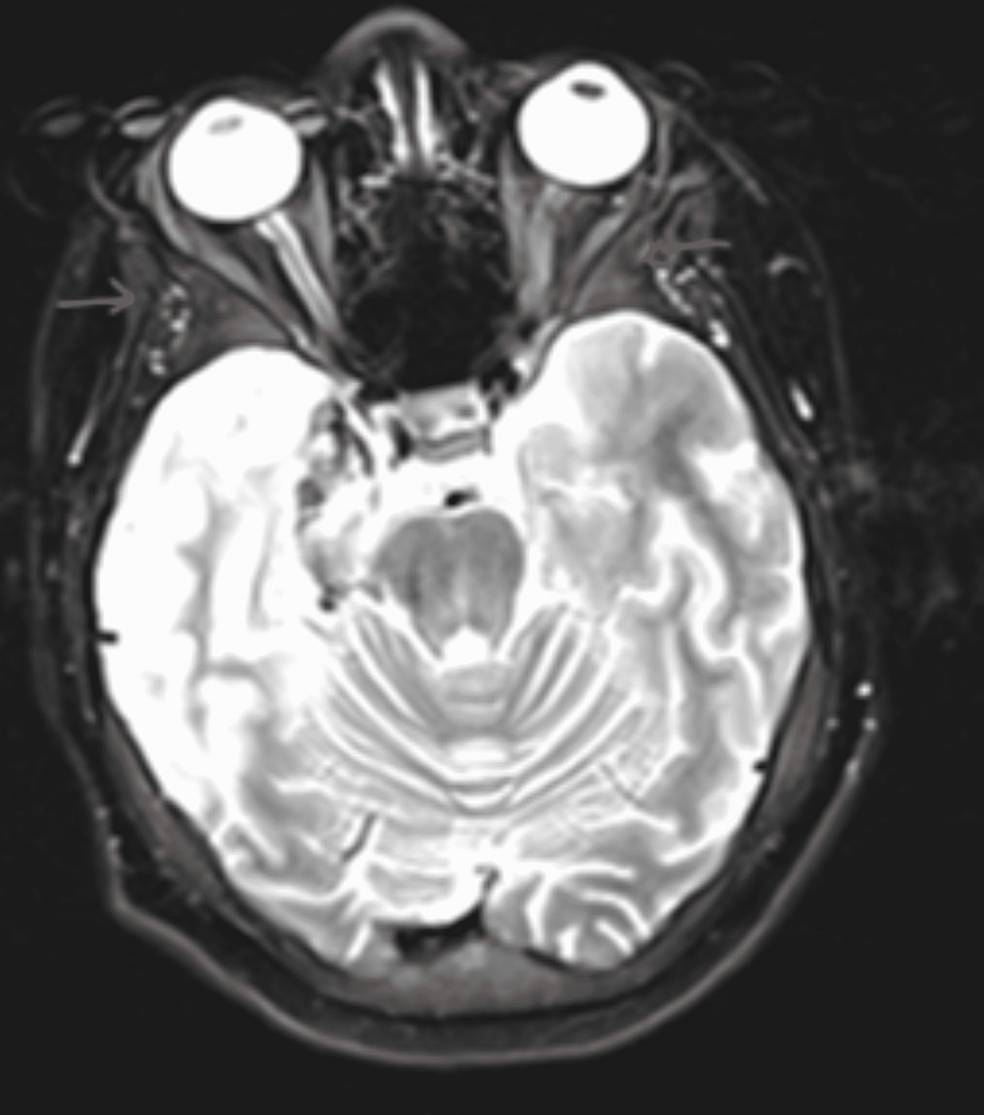
MRI brain with contrast focused on orbits, T2-weighted, axial view The axial view shows bilateral ocular globes and extra-ocular muscles that are normal.

**Figure 11:**
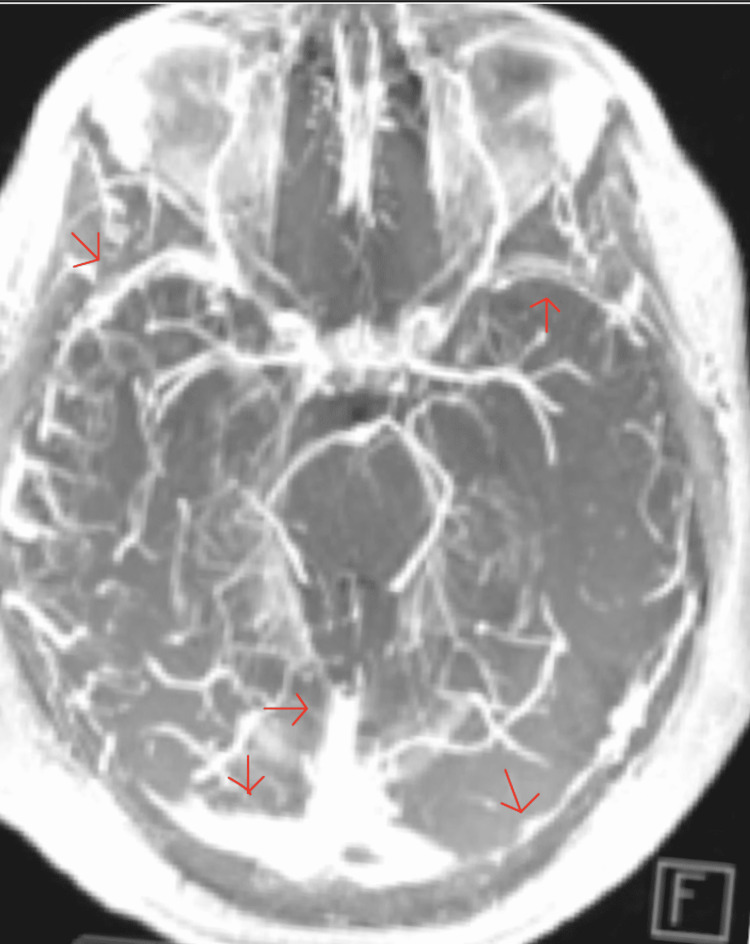
MRV of the brain Magnetic resonance venography  (MRV) of the brain shows patent dural venous sinuses (red arrows).

Two days prior to discharge the patient had a follow-up visit with ophthalmology that showed increased papilledema in the right eye. Initiation of Diamox and repeat brain imaging was recommended. The patient had a repeat MRI of the brain with and without contrast, along with an MRI of the orbits (Figure 9 and Figure 10) that showed stable cerebral edema and hemorrhagic transformation with unremarkable orbits with no signs of herniation and magnetic resonance venography (MRV) (Figure 11) that showed patent dural venous sinus. Repeat lumbar puncture two days prior to finishing the course of acyclovir showed WBC of 64/cumm (range 0-6/cumm) with a negative NAAT for HSV-1 and an opening pressure of 8 mmHg (seen above in Table 1). Diamox was discontinued at this time. After completing 21 days of IV acyclovir, the patient was discharged home with continued anti-epileptics and suppressive antiviral medications, hemodynamic stability, mild headaches, and improvement in vision and esotropia. 

Follow-up appointments with pediatric neurology showed normal repeat EEGs and significantly improved right parietal cerebral edema on repeat MRIs. The patient was kept on anti-epileptics and anti-viral (acyclovir) suppressive medications for one year. Two and six months after being discharged the patient was doing well, with minimal migraine-type headaches occurring once or twice a month, her vision was back to baseline, and she was able to continue going to school.

## Discussion

Herpes simplex virus meningoencephalitis has become the most common cause of sporadic fatal encephalitis in the United States. It results from an acute inflammation of mononuclear cells in infected tissue, most prominently found in the temporal lobes of adolescents and adults or diffusely in neonates. The source of infection is found in the central nervous system originating from a primary infection with the first herpes simplex virus subtype, although the second subtype has been known to cause encephalitis [1]. 

Herpes simplex viruses belong to the large, enveloped double-stranded DNA viruses. The first subtype traditionally involves the face and skin above the waist but there have been cases of genital involvement. The second subtype involves the genitalia and skin below the waist in usually sexually active adolescents. The primary infection is usually established as a neonate and then remains latent in the trigeminal nerves until a period of reactivation when the patient becomes symptomatic or asymptomatically sheds the virus. The incubation period for the infection to occur after the neonatal period ranges from two days to two2 weeks [2]. 

The transmission from an infected mother to a neonate usually happens during delivery and it occurs in approximately one to 3,000 to 20,000 vaginal births. However, women who experience a reactivation of HSV during the intrapartum period have only a 3% chance of transmitting the virus to the infant. Of those infected neonates, only 30% had mothers who were symptomatic with HSV-1, and the other 70% occurred with asymptomatic shedding of the virus after the primary episode of infection [3]. Neonatal HSV infections that occur in the intrapartum period or postnatally due to contact with lesions, usually occur in infants born prematurely or that have low birth weight. Congenital HSV infections occur in infants that are born with microcephaly, hydrocephalus, chorioretinitis, and vesicular skin lesions [3]. Neonatal HSV usually manifests in disseminated disease that involves the liver and lungs and in about three-fourths of the cases in the CNS [1], adrenals, skin, eye, or mouth [3]. If the neonatal infection is localized to the CNS, it can involve the skin, eyes, or mouth, or not involve the CNS. The diagnosis of HSV infection in neonates without skin manifestations is difficult but one needs to speculate if a neonate presents concerns for sepsis and there are negative bacterial cultures, severe liver dysfunction, coagulopathy, or suspected viral pneumonia then there is a chance for HSV-disseminated disease. Initial signs of HSV with or without skin lesions in neonates can occur between birth to six weeks but most present at about the first or second week of life and those that present with CNS disease present at about the second or third week of life [1]. 

According to studies performed by the National Institute of Allergy and Infectious Disease, one-third of HSE cases under the age of 18 are primary infections that may have found their access to the temporal lobe of the brain via the olfactory and/or trigeminal nerves. Herpes simplex encephalitis has been estimated to occur in one in every 250,000 to one in every 500,000 individuals a year, which in the United States would account for 10% to 20% of all encephalitic CNS viral infections [1]. Of these, about one-fifth of the cases occur in the pediatric population. In this population, they present with a wide range of symptoms and signs that include fever, mental status changes, personality changes, seizures, and focal neurological deficits. Untreated, it leads to a fulminant course which includes coma and ultimately death. 

The MRI of the brain with and without contrast is the most sensitive imaging modality to diagnose HSE [2]. Imaging studies will show an asymmetric hyperintense area on the T2-weighted series that indicates areas of swelling around the mesial-temporal and orbitofrontal lobes, which are in the anterior temporal lobes and insular cortex. The CT of the brain in early disease is not recommended since there is only a 50% chance of it being detected [1]. Later in the disease course, hemorrhagic transformation of the area can be seen. One study looked at the pediatric and adult population with confirmed HSE over a 12-year period, in which 22 of the 1748 encephalitis cases were pediatric cases. This study showed that neuroimaging in 95% (21) pediatric cases had abnormal MRI and 70% (14) had abnormal CT scans. The MRI in seven patients showed isolated temporal lobe involvement and in five patients there was a hemorrhagic component [4]. Along with MRI/CTs, lumbar punctures showed CSF studies with pleocytosis with a lymphocytic predominance. The CSF obtained is used to run polymerase chain reaction assays to detect HSV DNA, which is the diagnostic method of choice for this disease [2]. Another neuroimaging study that is performed after lumbar punctures and MRIs/CTs, especially if patients present with seizures are EEGs [3]. Of the 21 patients in the previously mentioned study, 15 had EEGs and of those, 13 patients (87%) were abnormal. The EEG findings ranged from diffuse multifocal slowing in eight patients (53%), periodic lateralized epileptiform discharges in two patients (13%), and/or temporal epileptiform activity in two patients (13%) [4]. 

The primary treatment for HSV meningoencephalitis starts with a 21-day course of IV acyclovir [1]. It is important that a repeat lumbar puncture with HSV PCR be performed close to the end of the 21-day IV course to determine if further treatment is needed [5]. As for the addition of steroids due to the increased cerebral edema, there has not been any study to prove that it is effective compared to a placebo group. Regarding long-term treatments for HSE, there was a randomized double-blinded clinical trial in 2012 that used 2 gm of valacyclovir three times a day for 90 days, after having completed the 21-day acyclovir IV course. This trial was done to determine if there was any improvement in neurological status with the use of a prophylaxis dose after an encephalitis event. The trial measured for any neurological impairment over three different periods throughout the course of a year using two different scales, namely the Mattis dementia rating scale (MDRS) and mini-mental state examination (MMSE). Overall, with appropriate treatment with 21 days of acyclovir, there was no statistical difference in the use of valacyclovir versus placebo for long-term prophylaxis. Of the 79 subjects, 84% of those using valacyclovir and 88% of those given a placebo, there was no or mild impairment at three months and 12 months using MDRS. The MMSE at day 90, showed that 90% of valacyclovir recipients and 86% of placebo recipients had no or mild impairment; at 12 months, the figures were 88% and 86%, respectively. This study notes that the key to preventing significant neurological impairment is prompt initiation of acyclovir therapy [6]. 

## Conclusions

Two months after being discharged, the patient was doing very well with minimal headache complaints occurring once or twice a month, her vision was back to baseline, and she was able to continue going to school. Meningoencephalitis caused by HSV is part of the CNS disorders that can arise from a primary or recurrent HSV infection and are associated with high morbidity and mortality. It is important to remember that prompt initiation of acyclovir treatment is key for long-term neurological outcomes and decreased risk of mortality from encephalitis. Lumbar punctures should not be delayed for imaging studies when there is a high suspicion of any type of meningitis to initiate timely, appropriate empiric treatments. Also, it is essential to go back to the basics of acute emergency patient care to stabilize patients for further investigation. In this case, it was to stop the patient from actively seizing to maintain her airway, breathing, and circulation. Most importantly, a careful review of past medical history may reveal neonatal infections and increase the suspicion of HSE in children of all ages presenting with headaches and seizures, with or without initial altered mental status. 
